# Binary MoS_2_ nanostructures as nanocarriers for amplification in multiplexed electrochemical immunosensing: simultaneous determination of B cell activation factor and proliferation-induced signal immunity-related cytokines

**DOI:** 10.1007/s00604-022-05250-4

**Published:** 2022-03-14

**Authors:** Beatriz Arévalo, Marina Blázquez-García, Alejandro Valverde, Verónica Serafín, Ana Montero-Calle, Guillermo Solís-Fernández, Rodrigo Barderas, Susana Campuzano, Paloma Yáñez-Sedeño, José M. Pingarrón

**Affiliations:** 1 Department of Analytical Chemistry, Faculty of Chemistry, Complutense University of Madrid, 28040 Madrid, Spain; 2 Chronic Disease Programme, UFIEC, Institute of Health Carlos III, 28220 MajadahondaMadrid, Spain

**Keywords:** Dual immunoplatform, BAFF, APRIL, MoS_2_/MWCNTs, Amperometry, Serum, SLE, CRC

## Abstract

A dual immunosensor is reported for the simultaneous determination of two important immunity-related cytokines: BAFF (B cell activation factor) and APRIL (a proliferation-induced signal). Sandwich-type immunoassays with specific antibodies (cAbs) and a strategy for signal amplification based on labelling the detection antibodies (dAbs) with binary MoS_2_/MWCNTs nanostructures and using horseradish peroxidase (HRP) were implemented. Amperometric detection was carried out at screen-printed dual carbon electrodes (SPdCEs) through the hydroquinone HQ/H_2_O_2_ system. The developed dual immunosensor provided limit of detection (LOD) of 0.08 and 0.06 ng mL^−1^ for BAFF and APRIL, respectively, and proved to be useful for the determination of both cytokines in cancer cell lysates and serum samples from patients diagnosed with autoimmune diseases and cancer. The obtained results agreed with those found using ELISA methodologies.

**Graphical abstract:**

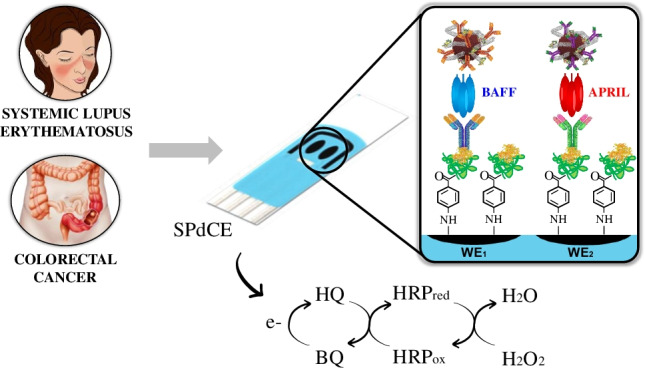

**Supplementary Information:**

The online version contains supplementary material available at 10.1007/s00604-022-05250-4.

## Introduction

BAFF (B cell activating factor) and APRIL (a proliferation-inducing ligand) are molecules similar to tumor necrosis factor mainly expressed on B lymphocytes that show structural similarities and interact with three receptors of the TNF family specifically and redundantly: BAFF-R joins BAFF, BCMA joins APRIL and also BAFF with weaker affinity, and TACI joins BAFF and APRIL equally well [1]. Both are type II membrane proteins, and the former is released after being cleaved at the furin protease site as a biologically active and soluble 17 kDa cytokine [2]. APRIL is also formulated as a membrane‐bound protein, which is broken down by furin as a soluble trimeric cytokine [3]. B cells play a pivotal role in autoimmunity not only by producing autoantibodies but also by modulating immune responses via the formation of cytokines and chemokines. The BAFF/APRIL system promotes B cell survival and differentiation, and therefore such cytokines are involved in the pathogenesis of autoimmune diseases. For instance, elevated blood or tissue levels of these biomolecules are frequently observed in patients of systemic lupus erythematosus (SLE) [4], lupus nephritis [5], Sjögren disease [6], or rheumatoid arthritis (RA) [7]. Regarding SLE, BAFF and APRIL serum levels correlate positively with disease activity and other markers, such as the level of serum anti-dsDNA antibodies. In addition, an elevated serum BAFF concentration (equal or greater than 2 ng mL^−1^) predicts the occurrence of moderate to severe SLE flares in patients receiving treatment [4]. On the other hand, increased serum levels of these two cytokines have also been associated with tumor growth and invasion in certain malignancies such as breast cancer [8].

Despite their importance for monitoring autoimmune diseases which affect large population, no immunosensors for these cytokines have been reported in the literature. Nevertheless, various colorimetric ELISA kits are commercially available for the single determination of BAFF or APRIL. These kits provide logarithmic calibrations over the pg mL^−1^ or ng mL^−1^ range and require assay times lasting around 4 h. Examples are the Quantikine ELISA Human BAFF/TNFSF13B Immunoassay with a claimed minimum detection dose (MDD = $\overline{x}\pm 2 \mathrm{s }$) of 6.44 pg mL^−1^ and dynamic range between 62.5 and 4000 pg mL^−1^ BAFF, and Human APRIL/TNFSF13 Quantikine ELISA Kit DAPR00 Immunoassay, in the range between 0.2 and 10 ng mL^−1^ and MDD = 0.015 ng mL^−1^ APRIL.

On the other hand, 2D materials, mainly graphene, have been widely used in the last years for technological applications. However, recently, transition metal dichalcogenides (TMDs) have replaced graphene in some applications due to their unique physico-chemical properties. Among TMDs, MoS_2_ is widely used in bioelectronics because of its biocompatibility, semiconductivity, and intrinsic peroxidase-like catalytic activity [9]. Electrochemical biosensors prepared with sheets [10, 11], nanoparticles [12], or MoS_2_ quantum dots [13] have been reported. Furthermore, the poor intrinsic conductivity of MoS_2_ has been improved by preparation of hybrids or composites with carbon nanomaterials to enhance the electron transfer reaction at the interface [14]. Various electrochemical biosensors involving hybrids of MoS_2_ and graphene oxide (GO) [15, 16] or carbon nanotubes (CNTs) [17] have been reported.

In this work, MoS_2_ composite materials with multi-walled carbon nanotubes (MoS_2_/MWCNTs), reduced graphene oxide (MoS_2_/rGO), and a mixture of both nanomaterials (MoS_2_/rGO/MWCNTs) have been synthesized to select the most appropriate to be used as carrier tag for signal amplification. Characterization of the resulting composite materials and a comparative evaluation of their electrochemical behavior led us to choose MoS_2_/MWCNTs, since it exhibited the highest pseudo-peroxidase activity and excellent electrochemical characteristics.

Accordingly, a rapid and highly sensitive dual immunosensor for the simultaneous amperometric determination of BAFF and APRIL at screen-printed dual carbon electrodes (SPdCEs) is presented for the first time in this paper. The specific capture antibodies were covalently immobilized by employing carbodiimide/hydroxysuccinimide chemistry on the surface of the modified working electrodes after electrochemically grafting the diazonium salt of 4-aminobenzoic acid (*p*-ABA), which resulted in covalent binding of the 4-carboxyl phenyl moieties on the carbon surfaces. Sandwich-type immunoassays were implemented using MoS_2_/MWCNTs(-HRP)-dAbs as carrier labels for signal amplification. In these nanocarriers the acid-treated MWCNTs provided the required functional groups to immobilize a large number of HRP and dAb molecules, and MoS_2_ contributed to improve sensitivity due to its pseudo-peroxidase activity. Amperometric measurements at *E*_app_ =  − 0.20 V vs. Ag pseudo-reference electrode in the presence of H_2_O_2_ and hydroquinone (HQ) were employed to follow the extension of affinity reactions. The developed dual immunosensor successfully tackled the determination of the two target cytokines in cancer cell lysates and serum samples from healthy subjects and patients diagnosed with SLE and colorectal cancer (CRC).

## Experimental

### Reagents, solutions and samples

Multi-walled carbon nanotubes (MWCNTs; *ϕ* 30 ± 15 nm, 95% purity) were supplied by NanoLab, Brighton, MA. Before use, CNTs were chemically shortened and carboxylated by treatment with nitric and sulfuric acids (v/v 1:3) under ultrasound for 5 h. The resulting product was centrifuged at 4000 rpm for 10 min and washed repeatedly with deionized water up to pH 7 and dried under nitrogen [18]. Graphene oxide (NIT.GO.M.140.10) from Nanoinnova Technologies was also used. Ammonium heptamolybdate ((NH_4_)_6_Mo_7_O_24_·4 H_2_O), thiourea, N-(3- dimethyl-aminopropyl)-N’-ethylcabodiimide (EDC), N-hydroxysulfosuccinimide (NHSS), biotin, streptavidin, *p*-aminobenzoic acid (*p*-ABA), sodium nitrite, peroxidase from horseradish (HRP) (Ref. P8250-25KU), streptavidin from *Streptomyces avidinii* (Strep) (Ref. S4762-5MG), hydroquinone (HQ), and hydrogen peroxide (H_2_O_2_, 30% w/v) were purchased from Sigma-Aldrich. Neutravidin (Neu) was from Thermo Fisher Scientific (Ref. 31,000). Sodium chloride, potassium chloride, sodium di-hydrogen phosphate, di-sodium hydrogen phosphate, and tris-hydroxymethyl-aminomethane-HCl (Tris–HCl) were from Scharlab. Anti-BAFF-biotin capture antibodies (b-cAb_BAFF_), anti-BAFF detection antibodies (dAb_BAFF_), and human BAFF/BLyS standard were from the Human BAFF/BLyS/TNFSF13B DuoSet ELISA from R&D Systems (Cat. No. DY124-05), and anti-APRIL-biotin capture antibodies (cAb_APRIL_), anti-APRIL detection antibodies (dAb_APRIL_), and human APRIL standard were from the Human APRIL/TNFSF13 DuoSet ELISA from R&D Systems (Cat. No. DY884B). Biotin was from Gerbu (Lot. 280,611).

Selectivity was checked against human hemoglobin (HB, Cat. No. H7379), albumin from human serum (HSA, Cat. No. A1653), IgG from human serum (hIgG, Ref: I2511), all of them from Sigma-Aldrich, human cadherin-17 from Origene (CDH-17, Ref: TP720740), human neurofilament-L from Cell Signaling Technology® (NfL, Cat. No. 99175), recombinant human tau-441 from BioLegend® (TAU), recombinant human TAR DNA-binding protein 43 from Abcam (TDP-43, Cat. No. ab224788), and human IL-13sRα2 (Ref: DY614) and human TNF (Ref: DY210) from R&D Systems.

Sera from CRC patients and from healthy individuals were obtained through the IdISSC biobank after approval of the Institutional Ethical Review Boards of the Hospital Clínico San Carlos and the Institute of Health Carlos III, and with the written informed consent of all participating individuals, whereas sera from SLE patients were purchased from Central BioHub. All serum samples were stored at − 80 °C, until use. All the experiments with these samples were performed accomplishing all the ethical issues and relevant guidelines and regulations of the involved institutions.

SW480 and SW620 (from the American Type Culture Collection (ATCC) cell repository) and KM12C, KM12SM, and KM12L4a (from I. Fidler’s laboratory, MD Anderson Cancer Center, Houston, TX) cells were grown and lysed as reported previously [19, 20].

Water purified by the Milli-Q purification system (18.2 MΩ cm) was used for the preparation of all solutions. The used buffers included B&W buffer pH 7.5 (0.01 mol L^−1^ Tris–HCl pH 7.5 containing 1 mmol L^−1^ EDTA and 2 mmol L^−1^ NaCl), 0.01 mol L^−1^ phosphate buffer saline solution (PBS) pH 7.5, and 0.05 mol L^−1^ phosphate buffer (PB) solution pH 6.0. The solutions used to perform the amperometric detection, 0.1 mol L^−1^ H_2_O_2_ and 0.1 mol L^−1^ HQ, were freshly prepared in phosphate buffer solution pH 6.0. 

The 1-Step™ Ultra TMB-ELISA Substrate Solution from Thermo Scientific™ (Cat. No. 34028) was also used.

### Apparatus and electrodes

All the amperometric measurements were made at room temperature using a CHI812B (CH Instruments, Inc.) potentiostat controlled by the CHI812B software. A μAutolab type III potentiostat (Ecochemie) controlled by FRA2 software electrochemical impedance spectroscopy (EIS) was employed for other electrochemical measurements.

Screen-printed carbon electrodes (SPCEs, DRP-110), with a 4 mm-Ø carbon working electrode, and dual SPCEs (DRP-X1110) consisted of two elliptic carbon working electrodes with a surface area of 5.6 mm^2^ were from Metrohm-DropSens. These electrodes include a carbon counter electrode and an Ag pseudo-reference electrode. The specific cable connectors (DRP-CAC and DRP-BICAC) used as interface between the SPCEs and dual SPCEs, respectively, and the potentiostat were also from Metrohm-DropSens. The measurements were made in stirred solutions using 10-mL glass electrochemical cells from Pobel. A Crison model Basic 20 + pH meter, a P-Selecta Ultrasons ultrasonic bath, a Heidolph Reax Top homogenizer for small samples, and an MPW-65R centrifuge from MPW (Med. Instruments) were also employed. Scanning electron microscopy (SEM) was made with a JEOM 7600 electron microscope operating at 5 kV. The transmission electron microscopy (TEM) characterization was performed using a JEM 2100 PLUS microscope operating at 100 kV. Raman analysis was performed in a NT-MDT NTEGRA Spectra spectrometer, equipped with a Solar TII MS5004i monochromator and an Andor iDUS DU-420 CCD detector. The excitation source is a laser of 532-nm wavelength and 22 mW. ELISA absorbance readings were made in a Sunrise™ Tecan microplate reader with the Magellan V 7.1 software.

## Procedures

### Preparation of MoS_2_/MWCNTs

The MoS_2_/MWCNTs composite was prepared by the hydrothermal route [21, 22]: 2.5 mg carboxylated MWCNTs (c-MWCNTs) suspended in 20 mL deionized water were ultrasonically stirred for 30 min to obtain a homogeneous dispersion. Then, 33 mg of ammonium heptamolybdate ((NH_4_)_6_Mo_7_O_24_·4 H_2_O) and 60 mg thiourea were added and, after stirring for 30 min at 800 rpm, the resulting solution was transferred to a Teflon-lined stainless-steel reactor and kept into an oven at 200 °C for 24 h. Once cooling down to room temperature (60 min approximately), the black precipitate was collected by centrifugation, washed three times with 10 mL ethanol, other three times with 10 mL water, and dried during 24 h at 37 °C. For comparison purposes, MoS_2_, MoS_2_/rGO, and MoS_2_/rGO/MWCNTs were also prepared by applying similar procedures to that described above with no carbon nanomaterials, with 3.0 mg GO, or with 3.0 mg GO plus 2.5 mg c-MWCNTs, respectively.

Stock solutions containing 1 mg mL^−1^ MoS_2_/MWCNTs were prepared by suspending the product in 10 mM PBS of pH 7.5 and stirring ultrasonically to obtain a homogeneous dispersion. Similarly, solutions from the other MoS_2_-based materials were prepared. The resulting dispersions were stored at room temperature in the dark.

### Preparation of MoS_2_/MWCNTs(-HRP)-dAbs

MoS_2_/MWCNTs(-HRP)-dAbs carrier tags were prepared through a similar protocol to that reported previously for the preparation of other nanocarriers involving MWCNTs hybrids [18, 23, 24]. Briefly, 500 μL of 0.1 mg mL^−1^ MoS_2_/MWCNTs was centrifuged at 14,000 rpm during 10 min and the remaining product was incubated in the darkness under continuous stirring for 6 h at room temperature with 200 μL of a 400 mM EDC and 100 mM NHSS mixture solution prepared in 100 mM PBS pH 7.4. The resulting solution was centrifuged at 14,000 rpm during 5 min and the product washed three times with 10 mM PBS pH 7.4. Activated MoS_2_/MWCNTs were conjugated during 24 h at 4 °C under continuous stirring with a mixture solution prepared in 100 mM PBS pH 7.4 containing 10 mg mL^−1^ HRP and 6 μg mL^−1^ dAb_BAFF_ to prepare MoS_2_/MWCNTs(-HRP)-dAb_BAFF_ conjugates. A similar procedure was used with 10 mg mL^−1^ HRP and 5 μg mL^−1^ dAb_APRIL_ to prepare MoS_2_/MWCNTs(-HRP)-dAb_APRIL_ labels. The as-prepared carriers were centrifuged at 14,000 rpm during 5 min, washed three times with 10 mM PBS pH 7.4, re-suspended in 500 μL of 100 mM PBST pH 7.4, and stored at 4 °C until their use.

### Preparation of the electrochemical scaffold

**Fig. 1:**
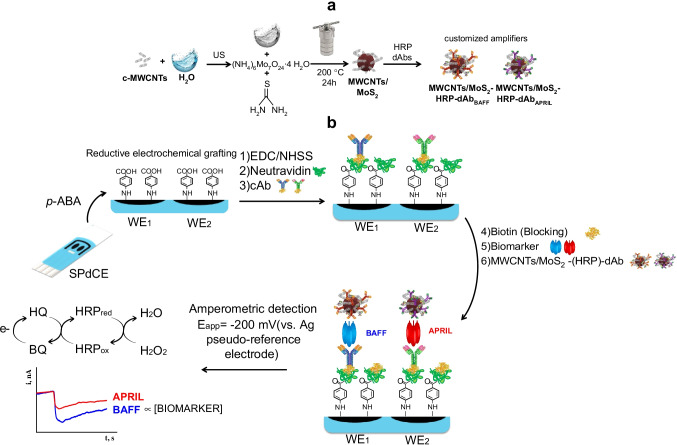
Schematic display of the different steps involved in the preparation of the MWCNTs/MoS_2_(-HRP)-dAb nanocarriers (**a**) and of the dual immunosensor constructed for the determination of BAFF and APRIL biomarkers using amperometric transduction (**b**)

The scheme depicted in Fig. 1 shows how both single and dual SPCEs were functionalized by reductive electrochemical grafting with *p*-ABA following the protocol reported previously [25] with slight modifications. In a first step, diazonium salt was prepared by adding dropwise 2 mM NaNO_2_ aqueous solution to a 1 mg mL^−1^ *p*-ABA solution prepared in 1 M HCl and cooling with ice (38 mL NaNO_2_ for each 2 mL *p*-ABA solution). The reaction was allowed proceeding for 7 min under stirring. Thereafter, each electrode was immersed into the diazonium salt solution and ten successive voltammetric cycles over the 0 to − 1.0 V range (ν = 200 mV s^−1^ vs. the Ag pseudo-reference electrode) were scanned. Finally, the modified SPCEs were washed thoroughly with Milli-Q water and dried at room temperature.

### Preparation of the immunosensors

The carboxylic groups of grafted SPCEs were activated by dropping 10 μL of a fresh 100 mM each EDC and NHSS mixture solution in 25 mM MES buffer of pH 5.0 on the working electrode surfaces and left to react for 30 min. After washing with the 25 mM MES buffer of pH 5.0, 3 μL of a 600 or 400 μg mL^−1^ Neu solution prepared in the same MES buffer was placed onto the SPdCE working electrodes for the preparation of BAFF and APRIL immunosensors, respectively, allowing incubation for 30 min. The electrode was rinsed with 10 mM PBS pH 7.4 and, subsequently, the biotinylated capture antibodies (cAb_BAFF_ or cAb_APRIL_) were immobilized by adding 3 μL of 100 ng mL^−1^ or 5 μg mL^−1^, respectively, prepared in 10 mM PBS pH 7.4 and incubating for 30 min. Then, a blocking step was carried out by adding 10 μL of a 2 mg mL^−1^ biotin solution prepared in the same PBS and incubated during 15 min. After washing with BB, 3 μL of the APRIL or BAFF standard solutions or the samples prepared in BB solution were placed onto each working electrode and incubated for 30 min. After rinsing with BB, 3 μL of the MoS_2_/MWCNTs(-HRP)-dAb bioconjugate suspension were dropped onto the electrode surface and incubated for 30 min. The resulting immunosensing platform was washed again with 10 mM PBS pH 7.4 and kept with a 25 μL drop of the same buffer until the electrochemical measurements were made. All incubation steps involved in the immunosensor fabrication were performed at room temperature in a humid environment to prevent drop evaporation.

### Amperometric measurements

The amperometric measurements were carried out by immersing the modified electrode into the measuring cell containing 10 mL of 50 mM PB pH 6.0 and 100 μL of a fresh solution of 100 mM HQ prepared in the same buffer. The measurements were carried out under stirring by applying − 0.20 V vs. the Ag pseudo-reference electrode. Once the background current was stabilized (~ 50 s), 50 μL of a 100 mM H_2_O_2_ solution prepared daily in 50 mM PB pH 6.0 were added and the variation in the cathodic current due to the HRP reduction of H_2_O_2_ mediated by HQ, reaching the steady state in ~ 100 s, was recorded. The analytical responses given through the text correspond to the difference between the steady state and the background currents. They are the mean values of three replicates, and the error bars displayed were estimated as three times the standard deviation of each set of replicates (*α* = 0.05).

Figure 1 shows a scheme of the steps involved in the preparation of the dual immunosensor as well as the reactions occurring in the amperometric detection.

### Statistical analysis

ROC analysis was performed using GraphPad Prism 9 ROC curve analysis functionality using Wilson/Brown method and a 95% confidence interval, and R (Version 4.1.1) using “Epi” and “ModelGood” packages.

## Results and discussion

### Characterization of MoS_2_ hybrids

**Fig. 2:**
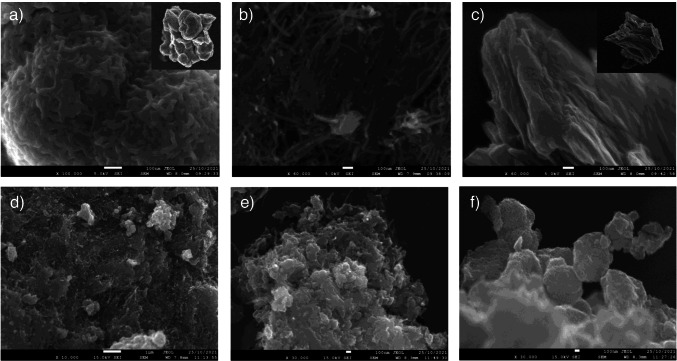
SEM images of (**a**) MoS_2_ (inset at higher magnification), (**b**) o-MWCNTs, (**c**) GO (inset at higher magnification), (**d**) MoS_2_/MWCNTs, (**e**) MoS_2_/rGO/MWCNTs, and (**f**) MoS_2_/GO

The morphology of MoS_2_ hybrids was characterized by scanning electron microscopy (SEM). Figure 2 shows some representative images of the bare nanomaterials (Fig. 2a–c), MoS_2_/MWCNTs (Fig. 2d), MoS_2_/rGO/MWCNTs (Fig. 2e), and MoS_2_/rGO (Fig. 2f) nanocomposites. Figure 2a displays two SEM images at different magnification levels of MoS_2_ obtained through the hydrothermal synthesis showing the formation of aggregates consisting of many sheets tightly stacked together [26]. MoS_2_/MWCNTs (Fig. 2d) shows a two-dimensional structure of carbon nanotubes (see Fig. 2b) with some decorating nanoparticles, presumably MoS_2_. Interconnections between MoS_2_ nanoparticles through carbon nanotubes and the high porosity suggest a good electrochemical behavior for this material. Regarding MoS_2_/rGO/MWCNTs (Fig. 2e), the SEM image shows a large amount of MoS_2_ nanoaggregates deposited onto the three-dimensional network formed by carbon nanomaterials [22]. However, when this morphology is compared with that of MoS_2_/rGO (Fig. 2f), a quite different structure is observed since MoS_2_ nanoparticles grew in a laminar way on the rGO sheets (see Fig. 2c), with no globular nanostructures, thus making difficult to distinguish between both materials. In summary, MoS_2_ composites prepared with carbon nanotubes mostly maintain the bare material structure providing rough surface morphology in the form of nanoaggregates, while in the presence of GO, the synthesized MoS_2_ appears as well-dispersed sheets on rGO substrate.

**Fig. 3:**
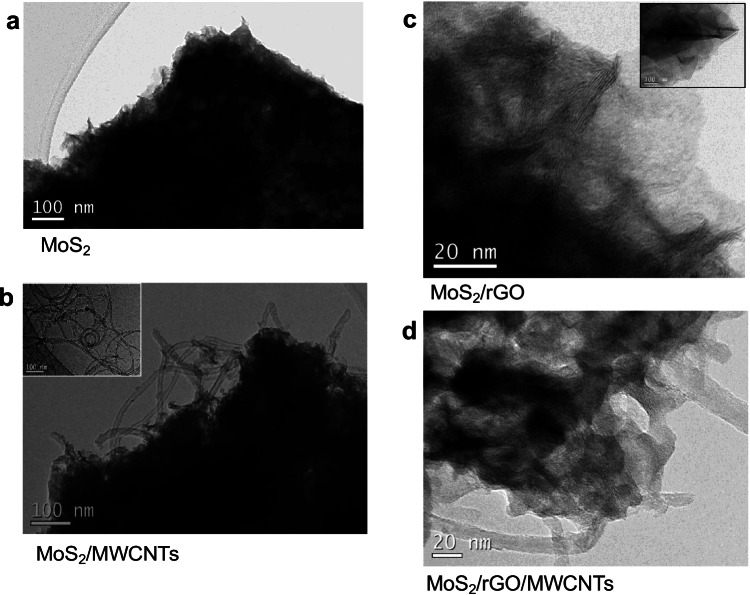
TEM images of: **a** MoS_2_, **b** MoS_2_/MWCNTs (inset: o-MWCNTs), **c** MoS_2_/GO (inset: GO), and **d** MoS_2_/rGO/MWCNTs

Transmission electron microscopy (TEM) was also used to investigate the structure of MoS_2_ nanocomposites. As Fig. 3 shows, bare MoS_2_ appears in the form of aggregated layers, this structure being preserved in the MoS_2_/MWCNTs nanocomposite, where some nanotubes can also be observed (Fig. 3b). In the MoS_2_/rGO composite (Fig. 3c), MoS_2_ nanosheets appear scattered onto rGO sheets. It has been reported that MoS_2_/rGO composites could acquire a 3D architecture caused by self-assembling during the hydrothermal process, in which reduction of GO can drive to partial overlapping or coalescing of the sheets [27]. Furthermore, a more complex structure showing MoS_2_ aggregates and some carbon nanotubes onto the rGO can be observed for MoS_2_/rGO/MWCNTs (Fig. 3d).

**Fig. 4:**
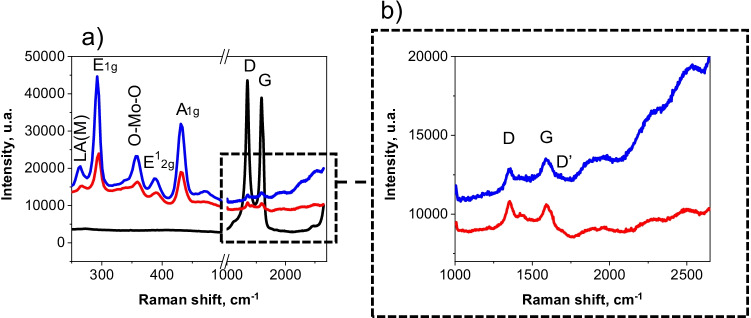
(**a**) Raman spectra and (**b**) zoom at high frequencies of MWCNTs (black), MoS_2_/MWCNTs (red), and MoS_2_/rGO/MWCNTs (blue) nanocomposites at *λ*_exc_ = 532 nm

Figure 4 compares the vibrational Raman spectra of MWCNTs, MoS_2_/MWCNTs, and MoS_2_/rGO/MWCNTs nanocomposites at a *λ*_exc_ of 532 nm. As can be seen, the spectra of the MoS_2_/MWCNTs and MoS_2_/rGO/MWCNTs nanomaterials displayed the vibration peak characteristic of MoS_2_: the E^1^_2g_, due to the in-plane opposing motions of S and Mo atoms, and the A_1g_ peak, which represents the out-of-plane relative motion of S atoms [28]. Moreover, a higher frequency shift was observed in the D and G bands of the MoS_2_/rGO/MWCNTs spectrum compared to that of MoS_2_/MWCNTs, indicating a substantial interaction between the MWCNTs, MoS_2_, and the rGO layers [29]. In addition, the peaks associated with LA(M), E_1g_, E^1^_2g_, and A_1g_ modes of MoS_2_, shifted from 267, 295, 392, and 431 cm^−1^ in the MoS_2_/MWCNTs to 264, 292, 387, and 430 cm^−1^ for the MoS_2_/rGO/MWCNTs. The band observed at 359 cm^−1^ in both MoS_2_ nanocomposites should be associated with O–Mo-O bending modes of bridging oxygen in MoO_3_ [30]. Furthermore, G, D, and D′ bands have been previously reported and attributed to defects in the processed MWCNTs [21].

**Fig. 5:**
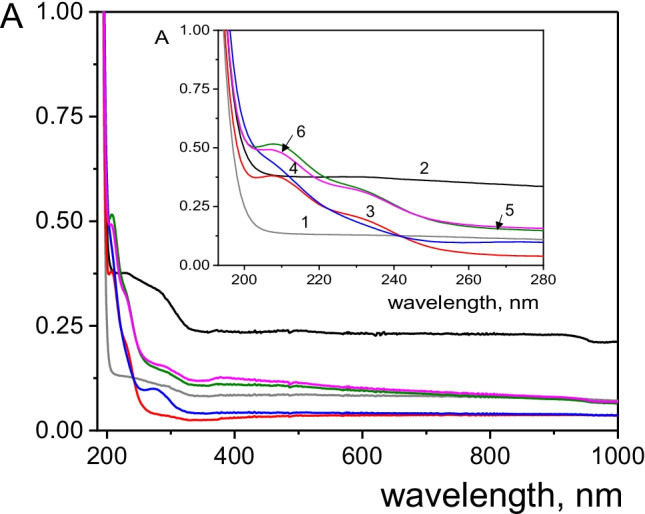
UV spectra of (1) GO, (2) o-MWCNTs, (3) MoS_2_, (4) MoS_2_/rGO, (5) MoS_2_/MWCNTs, and (6) MoS_2_/rGO/MWCNTs

UV–Vis spectroscopy was used to verify the formation of MoS_2_ hybrids (Fig. 5). Absorbance spectra from aqueous dispersions of the synthesized nanocomposites (30 μg mL^−1^) and the bare nanomaterials were recorded. No bands appeared in the range of scanned wavelengths for rGO (curve 1) or MWCNTs (curve 2). However, the MoS_2_ spectrum (curve 3) showed two absorption bands at 210 and 230 nm that also appeared in the MoS_2_/MWCNTs (curve 5) and MoS_2_/rGO/MWCNTs (curve 6) spectra. However, these bands are much poorly defined in the MoS_2_/rGO spectrum (curve 4) probably due to an encapsulation effect of MoS_2_ by rGO [16].

**Fig. 6:**
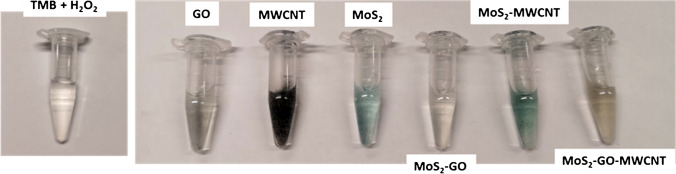
Photographs showing the peroxidase activity of MoS_2_ nanocomposites in 250 μL of the TMB/H_2_O_2_ ready to use commercial solution

In order to evaluate the peroxidase-like catalytic activity of the MoS_2_-based nanomaterials, the TMB/H_2_O_2_ system was employed as substrate. Figure 6 shows as the colorless solution developed a clearly and almost immediate visible blue color corresponding to the oxidized TMB in the presence of MoS_2_/MWCNTs. The blue color is due to the formation of a radical in the decomposition of hydrogen peroxide, which acts as an oxidant [31]. This same behavior was observed using other peroxidase substrates, OPD (o-phenylenediamine) and ABTS. These results show the higher ability of MoS_2_ to exhibit its intrinsic properties in composites prepared without rGO, due to the absence of encapsulation that occurs in the presence of flat graphene sheets [16]

### Electrochemical characterization of MoS_2_ nanomaterials

**Fig. 7:**
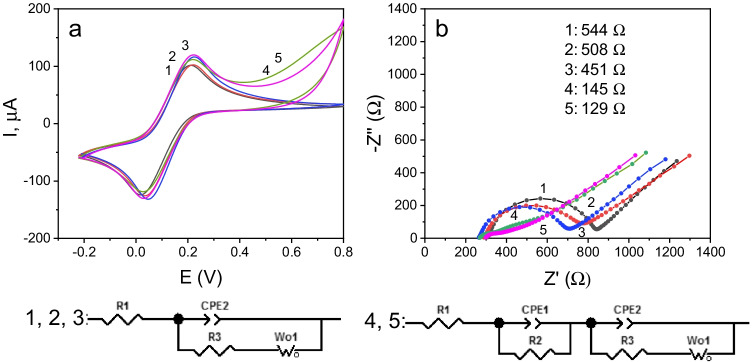
Cyclic voltammograms (**a**) and Nyquist plots (**b**) recorded for 5 mM [Fe(CN)_6_]^−3/−4^ in 0.1 M KCl solutions for (1) bare SPCE, (2) MoS_2_/SPCE, (3) MoS_2_/GO/SPCE, (4) MoS_2_/MWCNTs/SPCE, and (5) MoS_2_/GO/MWCNTs/SPCE. **a** *ν* = 50 mV s^−1^ and **b** range of frequencies: 10^5^–0.04 Hz; open circuit. The equivalent circuits used to adjust the experimental results are shown below

SPCEs were modified by dropping 5 μL of the as-prepared dispersions of MoS_2_ nanomaterials onto the working electrode surfaces and allowing drying. Then, cyclic voltammograms (CVs) and electrochemical impedance spectra (EIS) from 5 mM [Fe(CN)_6_]^−3/−4^ in 0.1 M KCl solutions were recorded (Fig. 7). As it can be seen, no significant voltammetric differences were apparent for all the electrodes, with similar anodic and cathodic *i*_*p*_, *E*_*p*_, and Δ*E* values in the electroactivity range of the redox probe. However, Nyquist spectra exhibited significantly lower electron transfer resistance (*R*_CT_) values for the electrodes modified with carbon nanotubes/MoS_2_ hybrids (MoS_2_/MWCNTs and MoS_2_/rGO/MWCNTs). This behavior can be attributed to a better conductivity of these composites due to the presence of carbon nanotubes, since such behavior was not observed for the electrode modified with MoS_2_ and rGO. Probably, as SEM and TEM images suggested, the carbon nanotube hybrids possess a more porous nanostructure that increases the specific surface of the electrode providing more active sites for target molecules. The larger differences observed using EIS compared with CV are probably due to the different rationale and mode of application of the two techniques. Whereas cyclic voltammetry involves large potential perturbations, in EIS the system is only infinitesimally perturbed with respect to the steady state.

### Optimization of the experimental variables involved in the preparation of the dual immunosensor

According to the scheme depicted in Fig. 1, the SPdCE was functionalized by electrografting with *p*-ABA, and the modified electrodes (*p*-ABA/SPdCE) were activated with EDC/NHSS for the covalent immobilization of Neu (Neu-Phe-SPdCE). Then, the biotinylated capture antibodies, cAb_BAFF_ and cAb_APRIL_, were immobilized on the respective working electrode and a sandwich configuration for each biomarker was implemented with MoS_2_/MWCNTs(-HRP)-dAb_BAFF_ or MoS_2_/MWCNTs(-HRP)-dAb_APRIL_ carrier tags.

The effect of the experimental variables involved in the preparation of MoS_2_/MWCNTs(-HRP)-dAb_BAFF_ (or -dAb_APRIL_) carrier tags and the cAb_BAFF_-Neu-Phe-SPdCE (or cAb_APRIL_-) bioelectrodes, on the amperometric responses provided by the resulting MoS_2_/MWCNTs(-HRP)-dAb_BAFF_-BAFF-cAb_BAFF_-Neu-Phe-SPdCE or MoS_2_/MWCNTs(-HRP)-dAb_APRIL_-APRIL-cAb_APRIL_-Neu-Phe-SPdCE, was evaluated. Larger ratio between the currents measured with the as-prepared immunosensors in the presence (S) of 5 ng mL^−1^ or 4 ng mL^−1^ BAFF or APRIL standards, respectively, or in the absence (B) of the target compounds was taken as the selection criterion for each tested variable. The optimization studies implied the evaluation of (a) MoS_2_/MWCNTs(-HRP)-dAb_BAFF_ (or -dAb_APRIL_) loadings, and those of dAb_BAFF_ (or dAb_APRIL_) and HRP onto the MoS_2_/MWCNTs nanocomposite; (b) selection between Strep or Neu binding protein immobilized onto Phe-SPdCEs; (c) loading and incubation time of Neu onto Phe-SPdCEs; (d) concentration and incubation time of biotinylated capture antibodies (cAbs) onto Neu-Phe-SPdCEs; (e) type, concentration, and incubation time of the blocking agent; (f) incubation time of BAFF or APRIL cytokines onto cAb-Neu-Phe-SPdCEs; and (g) incubation time of the corresponding nanocarrier tag onto BAFF-cAb_BAFF_-Neu-Phe-SPdCE or APRIL-cAb_APRIL_-Neu-Phe-SPdCE. The results of these studies are shown in the Supplementary Material (Figs. S1–S5) and summarized in Table 1. Moreover, the experimental conditions used for the modification of SPdCEs by grafting from the electrochemically generated *p*-ABA cation radical were the same as optimized previously [32]. The detection potential of − 0.20 V vs. Ag pseudo-reference electrode was also previously selected for the same catalytic system [33].

**Table 1:** Optimization of the experimental variables affecting the preparation and functioning of the dual immunosensor developed for the simultaneous determination of BAFF and APRIL cytokines

	BAFF		APRIL	
Variable	Tested range	Selected value	Tested range	Selected value
MWCNTs/MoS_2_ loading, mg mL^−1^	0.05–0.4	0.1	0.05–0.4	0.2
dAb loading, µg mL^−1^	0–9	6	0–20	5
HRP loading, mg mL^−1^	0.25–2	1	0.5–3	1
Affinity	Strep or Neu	Neu	Strep or Neu	Neu
Neutravidin loading, µg mL^−1^	0–800	600	0–800	400
Neutravidin incubation time, min	15–60	15	15–60	30
cAb, ng mL^−1^ (BAFF); µg mL^−1^ (APRIL)	0–500	100	0–10	5
cAb incubation time, min	15–60	30	5–60	15
Blocking mixture^*^	A–D	D	A–D	D
Biotin loading, mg mL^−1^	1–5	2	1–5	2
Biotin incubation time, min	15–60	15	15–60	15
Target protein incubation time, min	15–60	30	15–60	30
MoS_2_/MWCNTs(-HRP)-dAb incubation time, min	15–60	30	10–60	15

^*^(A) 2 mg mL^−1^ biotin, (B) 2% casein + 2 mg mL^−1^ biotin, (C) 2% BSA, and (D) 2% casein + 1% BSA + 3 mg mL^−1^ biotin.

### Electrochemical characterization of the immunosensors

The stepwise immunosensors’ preparation were monitored by EIS using 5 mM Fe(CN)_6_^4−/3−^ as redox probe. Figures S6a-d shows representative Nyquist plots and the equivalent circuits for the modified SPCE and the corresponding immunosensors. As can be seen, after modification of the SPCE with *p*-ABA there was a large increase in the electron transfer resistance, *R*_CT_, from 972 to 3862 Ω (Fig. S6a) because of electrostatic repulsion between the anionic redox probe and the surface-confined − COO^−^ groups of the modified electrode surface at the measuring pH. On the contrary, there was a drastic decrease in the *R*_CT_ value (not shown) after activation with EDC/NHSS due to the neutralization of these − COO^−^ groups. Then, the *R*_CT_ value remained relatively low after immobilization of Neu protein (2015 Ω and 1809 Ω, green curves in Figs. S6a and c, respectively). The impedance spectra corresponding to the immobilization of the immunoreagents are displayed in Figure S6b and d. As expected, an increase in the electron transfer resistance occurred upon immobilization of the biotinylated capture antibodies (cAb_BAFF_ or cAb_APRIL_), with *R*_CT_ values of 2196 Ω (BAFF) and 2076 Ω (APRIL), due to the resistance to electron transfer produced by the partially insulating layer created by these biomolecules on the electrode surface. In the case of the BAFF immunosensor (blue curve in Fig. S6b), the Nyquist diagram is somewhat distorted, while two semicircles are clearly observed in the APRIL spectrum (blue curve in Fig. S6d). This behavior can be attributed to the existence of two distinct layers on the surface of the electrode which is more evident in the case of APRIL because of the larger concentration of capture antibody (5 μg mL^−1^ vs. 100 ng mL^−1^). In both cases, the resistance increased after incubation with the antigen solutions (brown curves in Figs. S6c and d) because of hindered interfacial electron transfer by the immobilized proteins, with *R*_CT_ values of 2326 Ω (BAFF) and 2368 Ω (APRIL). However, a remarkable decrease was observed upon incubation with the MoS_2_/MWCNTs(-HRP)-dAb nanocarrier tag (purple curves in Figs. S6c and d), with *R*_CT (BAFF)_ = 2225 Ω and R_CT (APRIL)_ = 2187, which is most likely due to the higher conductivity of the resulting surface.

The obtained Nyquist plots can be defined by two different equivalent circuits as shown in Figure S6. The spectra of Figures S6a and c fitted well with the R1(C2[R3W1]) circuit, whereas the spectra in Figures S6b and d should be explained by a more complex scheme with at least two RC semi-circuits. The *R*_CT2_ value, corresponding to the second semicircle of the Nyquist plot, represented the electron transfer across the immobilized substances including biotinylated capture antibodies, the antigens, and the MoS_2_/MWCNTs(-HRP)-dAb nanocarrier tag.

### Analytical characteristics for the simultaneous determination of BAFF and APRIL

**Fig. 8:**
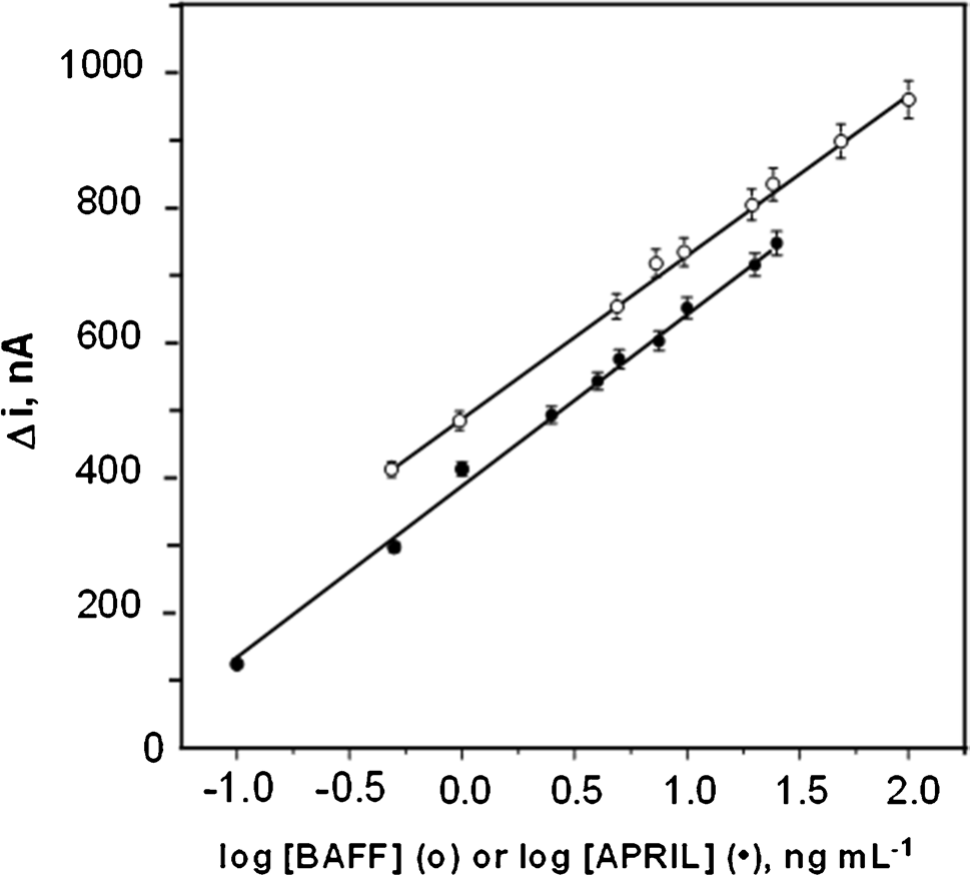
Calibration plots constructed with the dual immunosensor for BAFF (white points) and APRIL (black points) using MoS_2_/MWCNTs(-HRP)-dAb as carrier tags for the amperometric measurements at − 0.20 V vs. Ag pseudo-reference electrode. Other working conditions are summarized in Table 1

Figure 8 shows the calibration plots obtained with the dual immunosensor for BAFF and APRIL standard solutions. The relationships between currents (Δ*i*) and the logarithm of the biomarker concentration provide linear ranges from 0.24 to 120 ng mL^−1^ (*r*^2^ = 0.999) (BAFF) and 0.19 to 25 ng mL^−1^ (*r*^2^ = 0.997) (APRIL), fitting the equations: Δ*i*, nA = (244 ± 3) log [BAFF, ng mL^−1^] + (487 ± 3) and Δ*i*, nA = (258 ± 5) log [APRIL, ng mL^−1^] + (389 ± 4). These calibration data were compared with those obtained by constructing calibrations using MWCNTs(-HRP)-dAb, i.e., without MoS_2_ incorporated to the carbon nanotubes, or HRP-IgG-dAb, i.e., without MoS_2_ and MWCNTs.

In the first case, remarkably lower slope values, 86 ± 7 nA per decade of concentration (BAFF) and 179 ± 8 nA per decade of concentration (APRIL), were found over the same dynamic ranges with *r*^2^ = 0.991 and 0.993, respectively. The higher sensitivity achieved with the bioplatforms constructed with carrier tags involving MoS_2_ nanoparticles highlights their favorable contribution to enhance conductivity and their intrinsic pseudo-peroxidase activity that reinforces that of HRP. Furthermore, in absence of MoS_2_ and carbon nanotubes, the slope values are even lower, 39 ± 3 nA per decade of concentration (BAFF) (*r*^2^ = 0.990) and 140 ± 6 nA per decade of concentration (APRIL) (*r*^2^ = 0.994), due to the loss of the large specific surface area provided by carbon nanotubes to immobilize large amounts of detection antibodies, HRP, and MoS_2_ nanoparticles, and to promote fast and efficient electron transfer.

Limit of detection, LOD (0.08 ng mL^−1^ BAFF and 0.06 ng mL^−1^ APRIL), and limit of quantification, LQ (0.26 ng mL^−1^ BAFF and 0.19 ng mL^−1^ APRIL) values, were calculated according to the 3 *s*_*b*_/*m* and 10 *s*_*b*_/*m* criteria, where *s*_*b*_ was estimated as the standard deviation for ten amperometric measurements without target protein and *m* was the slope of the corresponding standard calibration plot (Fig. 8). The reproducibility of the measurements provided by the immunoplatforms was assessed by comparing the amperometric signals provided for 10 ng mL^−1^ of BAFF or 2.5 ng mL^−1^ of APRIL standards with 5 different immunosensors prepared in the same manner on both the same day (relative standard deviation, RSD, values of 2.9 and 2.4%, respectively) and different days (RSD of 3.6 and 3.3%, respectively). These results confirm the good reproducibility of the protocols involved both in the preparation of the immunosensors and in the amperometric measurements.

These analytical characteristics are compared with those claimed for the commercial ELISA kits. The kits that use the same immunoreagents (see “Experimental” section) provide logarithmic calibration plots with linear ranges from 39.1 to 2500 pg mL^−1^ BAFF and from 31.2 to 2000 pg mL^−1^ APRIL. Although these ranges start from lower concentrations, the immunoplatforms cover larger ranges. In addition, the kit protocols do not inform about the detection limits or precision levels. Other kits from the same company (see “Introduction” section) provide linear ranges between 62.5 and 4000 pg mL^−1^ BAFF with a minimum detection dose (MDD = $\overline{x}\pm 2 \mathrm{s }$) of 6.44 pg mL^−1^, and between 0.2 and 10 ng mL^−1^ APRIL with MDD = 0.015 ng mL^−1^. RSD values range between 3.4 and 11.6% (BAFF) and 3.8 and 8.5% (APRIL) for intra-assays and inter-assays, respectively. All these immunoassays require 4 h 30 min to be implemented. Therefore, it is worth highlighting the lower RSD values obtained with the dual immunosensor, as well as the shorter assay time. Indeed, the method reported in this paper allows the simultaneous determination of both cytokines in approximately 1 h 30 min counting since the cAbs were immobilized (similarly to that reported for the ELISA methods) onto the SPdCEs, i.e., in a three times shorter assay time than that required for the single determination of one biomarker by ELISA. In addition, the achieved sensitivities for both cytokines are perfectly adequate for the analysis of clinical samples where the expected concentrations are in the ng mL^−1^ range. So, levels in serum of healthy patients of 0.8 ng mL^−1^ BAFF [34] and 4 ng mL^−1^ APRIL [35] have been reported.

In addition, the stability of both immunoplatforms (cAb_BAFF/APRIL_-Neu-Phe-SPdCE, stored in a humid chamber) and bionanoconjugates (MWCNTs/MoS_2_-(HRP)-dAb_BAFF/APRIL_ resuspended in PBST) stored after their preparation at 4 °C was assessed by comparing the amperometric responses they provided in the absence and presence of BAFF (10 ng mL^−1^) or APRIL (4 ng mL^−1^) standards. According to these studies, the prepared bionanoconjugates were stable for at least 3 months (Figure S7a) and the immunoplatforms during 33 days (Fig. 7Sb), providing during these periods amperometric responses included within the limits of control set at ± 3 times the standard deviation of three measurements performed on the day of their preparation (day 0).

### Selectivity

**Fig. 9:**
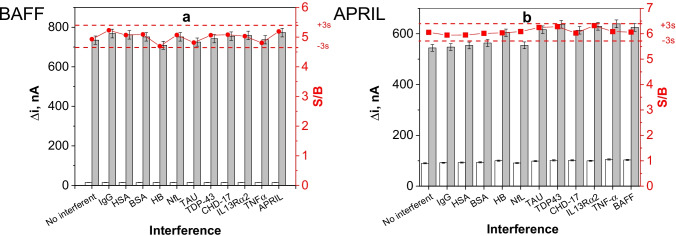
Amperometric responses measured with the dual immunosensor for 0- (white bar) and 10-ng mL^−1^ BAFF or 4-ng mL^−1^ APRIL (grey bar) standards prepared in absence and in the presence of 1 mg mL^−1^ hIgG, 50 mg mL^−1^ HSA, 5 mg mL^−1^ BSA, 5 mg mL^−1^ HB, 30 pg mL^−1^ NfL, 5 pg mL^−1^ TAU, 1 ng mL^−1^ TDP-43, 500 ng mL^−1^ CDH-17, 10 ng mL^−1^ IL13Rα2, 100 pg mL^−1^ TNFα, 4 ng mL^−1^ APRIL, or 1 ng mL^−1^ BAFF

The selectivity of the dual immunoplatform was checked by comparison of the currents measured for 0- and 10-ng mL^−1^ BAFF or 0- and 4-ng mL^−1^ APRIL standards, in absence or in the presence of various non-target proteins coexisting in serum as well as other biomarkers for autoimmune diseases and cancer at the concentration they found in healthy individuals. The results displayed in Fig. 9 proved that there were no significant differences in the S/B ratios for all proteins tested, falling in all cases within the range of ± 3 × standard deviation of the current measured in the absence of interferent (the corresponding Δi values, in nA, are given in Table S1 of the Supplementary Information).

The possible cross-talking between the two close bioelectrodes, WE1 (BAFF immunosensing surface) and WE2 (APRIL immunosensing surface), at the dual SPCE was evaluated. Figure S8 in the Supplementary Information compares the amperometric currents measured with the dual immunosensor in solutions containing varying concentrations of BAFF and APRIL standards, ensuring the absence of cross-talking between immunosensing surfaces due to the presence of the non-target cytokine.

### Application to the analysis of cancer cell lysates and serum samples

The developed immunosensors were applied to the analysis of the two target cytokines in CRC cell lysates with different metastatic potential and in serum samples from healthy individuals and from patients diagnosed with SLE and CRC.

Table S2 in the Supplementary Information shows a statistical comparison of the slope values of the calibration plots constructed for both analytes using standards prepared in the different matrices. As it can be seen, no apparent matrix effect was found for the determination of the two biomarkers for ten-fold diluted serum samples and 0.5 µg of cell extract.

Accordingly, the determination of both cytokines in the different analyzed samples was carried out simply by interpolation of the amperometric responses provided by the immunosensors in the calibration plots constructed with standard solutions of the cytokines (Fig. 8). The results obtained as well as those provided by the ELISA methodologies for the single determination of each cytokine are summarized in Table 2.

**Table 2:** Concentrations of BAFF and APRIL (in ng mL^−1^) determined using the developed dual immunosensors and the ELISA methods for their single determination in cell extracts and serum samples

		BAFF			APRIL		
Sample	Cell/individual	Immunosensor^*^	ELISA^*^	*t*_exp_^**^	Immunosensor^*^	ELISA^*^	*t*_exp_^**^
Cell extracts	KM12C	1.7 ± 0.1; 2.5	1.9 ± 0.3; 5.4	0.314	1.7 ± 0.1; 3.0	1.7 ± 0.3; 6.1	0.001
	KM12SM	5.00 ± 0.03; 2.4	5.3 ± 0.6; 4.8	0.348	7.0 ± 0.6; 3.4	8 ± 1; 7.5	0.828
	KM12L4a	3.2 ± 0.2; 2.0	3.1 ± 0.3; 4.5	0.070	4.5 ± 0.4; 3.5	4.2 ± 0.5; 4.6	0.324
	SW480	0.90 ± 0.07; 3.2	1.1 ± 0.2; 8.0	0.275	0.81 ± 0.05; 2.4	0.8 ± 0.1; 5.1	0.043
	SW620	2.6 ± 0.2; 2.5	2.8 ± 0.4; 6.1	0.309	3.1 ± 0.2; 2.9	3.4 ± 0.5; 6.2	0.385
Serum	Healthy	0.45 ± 0.05; 3.6	0.49 ± 0.09; 7.1	0.057	2.2 ± 0.1; 2.1	2.2 ± 0.2; 3.5	0.026
		0.29 ± 0.03; 3.8	0.29 ± 0.05; 6.9	0.011	1.70 ± 0.08; 1.9	1.7 ± 0.2; 4.5	0.051
		0.32 ± 0.03; 3.5	0.34 ± 0.04; 5.2	0.004	2.2 ± 0.2; 1.5	2.2 ± 0.3; 5.3	0.050
		0.22 ± 0.02; 3.8	0.23 ± 0.04; 7.7	0.005	1.70 ± 0.06; 1.4	1.6 ± 0.3; 7.3	0.149
		0.37 ± 0.04; 4.8	0.39 ± 0.05; 5.0	0.027	1.9 ± 0.2; 3.6	2.2 ± 0.3; 5.3	0.395
		0.14 ± 0.05; 2.8	0.12 ± 0.02; 7.0	0.015	1.8 ± 0.1; 3.3	1.8 ± 0.3; 6.4	0.007
		0.26 ± 0.02; 3.6	0.30 ± 0.06; 7.9	0.043	2.0 ± 0.2; 3.6	1.9 ± 0.3; 6.1	0.052
		0.57 ± 0.07; 4.8	0.7 ± 0.1; 6.2	0.110	1.6 ± 0.2; 4.5	1.6 ± 0.3; 7.4	0.162
	SLE	1.9 ± 0.2; 4.4	1.9 ± 0.3; 5.6	0.027	6.7 ± 0.4; 2.3	7.2 ± 0.8; 4.4	0.573
		1.20 ± 0.09; 3.1	1.2 ± 0.2; 5.9	0.065	6.4 ± 0.5; 3.2	6 ± 1; 6.6	1.021
		2.0 ± 0.1; 2.8	2.1 ± 0.3; 6.0	0.125	7.0 ± 0.3; 1.8	6 ± 1; 6.8	0.703
		1.4 ± 0.1; 3.5	1.4 ± 0.2; 6.6	0.012	7.0 ± 0.3; 2.0	6.3 ± 0.6; 3.9	0.893
	CRC	3.1 ± 0.2; 5.0	3.6 ± 0.6; 6.4	0.652	7.5 ± 0.6; 3.2	7.3 ± 0.7; 3.7	0.233
		4.3 ± 0.8; 3.1	4.2 ± 0.7; 6.6	0.044	7.8 ± 0.6; 3.4	8.2 ± 0.6; 3.0	0.449
		2.9 ± 0.2; 3.3	3.1 ± 0.5; 6.0	0.321	4.8 ± 0.3; 2.2	4.8 ± 0.5; 4.0	0.002
		4.5 ± 0.4; 3.3	4.9 ± 0.6; 4.8	0.518	8.1 ± 0.7; 3.6	7.9 ± 0.7; 3.4	0.148

^*^Mean value ± *t* × *s*/√*n*; RSD, %, *n* = 3, *α* = 0.05.

^**^t_exp_ < *t*_tab_ of 4.303 (*n* = 3, *α* = 0.05).

The obtained results revealed a higher expression of BAFF and APRIL in cells with higher metastatic potential (KM12SM, KM12L4a, and SW620), in agreement with the role played by these cytokines in the tumorigenesis and metastasis of CRC [36–38].

**Fig. 10:**
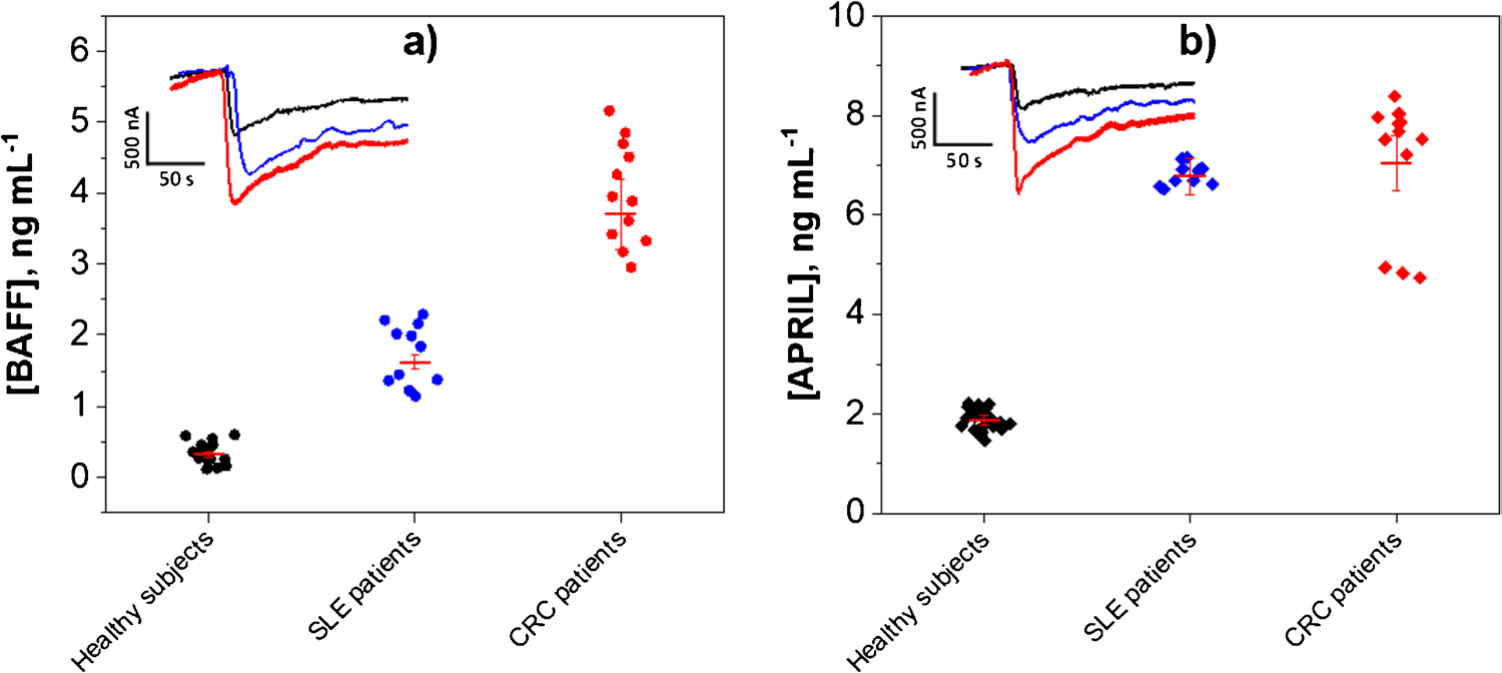
BAFF (**a**) and APRIL (**b**) concentrations determined with the dual immunosensors for serum samples grouped into pools of healthy individuals and SLE and CRC patients. Real amperograms obtained for representative serum samples of a healthy individual and patients diagnosed with SLE and CRC are displayed

Similarly, the results obtained in serum samples demonstrate the significantly higher expression of both cytokines in patients with SLE and CRC compared to healthy individuals. Figure 10 displays the results by patient group showing that it is possible to clearly discriminate patients with these important pathologies from healthy individuals by interrogating these cytokines. It is important to note that, in general, the serum level of APRIL is larger than that of BAFF in the three groups of analyzed patients.

The obtained results agree with those reported in the literature on the hyperexpression of these cytokines for SLE [39–42] and CRC [37, 43–46] patients.

In addition, the BAFF concentrations found agree with those reported by other authors for SLE patients and healthy individuals (1.47 ± 1.54 vs. 0.517 ± 0.18 ng mL^−1^ [40]) and (3.19 ± 4.26 vs. 0.97 ± 0.21 ng mL^−1^ [41]) as well as with the cut-off value established at 1429.4 pg mL^−1^ [42].

**Fig. 11:**
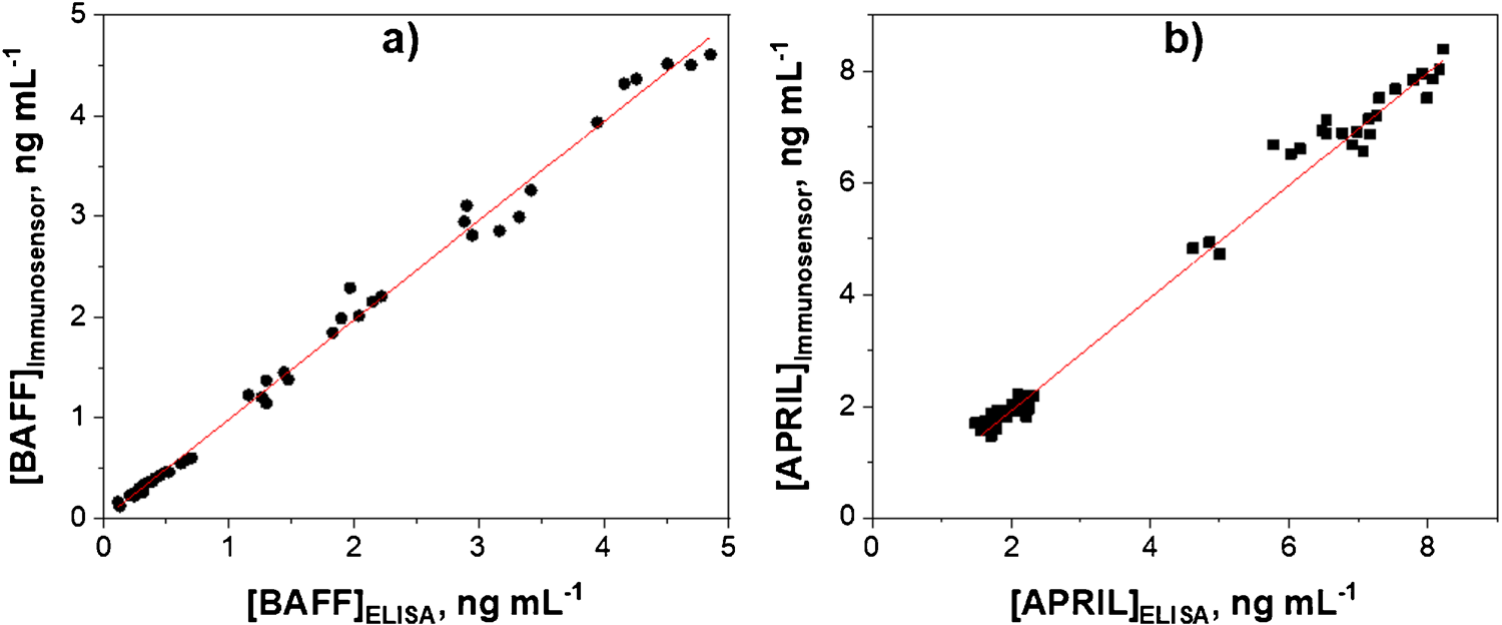
Correlation plots between the BAFF (**a**) and APRIL (**b**) concentrations determined with the developed dual inmunosensors and the ELISA methodologies for both the analyzed cellular lysates and serum samples. The three replicates made for each sample are included in the plots

The statistical analysis of the results provided by the developed immunoplatforms against those obtained by the ELISA methods for the single determination of both cytokines showed an excellent correlation (Fig. 11), thus confirming the accuracy of the results provided by the dual immunosensor.

To further deepen in the potential of the developed bioplatforms for diagnosing patients with SLE and CRC by amperometric determination of the two target cytokines, and to establish the best cut-off values that allow reliably discriminating between the three groups of patients analyzed, the results obtained were plotted on the ROC curves shown in Figure S9. According to them for the comparison between healthy individuals and SLE or CRC patients the area under the curve was 100%. When comparing SLE patients to healthy, a maximum cut-off with 100% specificity and 100% sensitivity was achieved for a concentration of BAFF equal to 0.885 ng mL^−1^. Similarly, CRC patients versus healthy individual comparison had a best cut-off with 100% specificity and also 100% sensitivity for 1.735 ng mL^−1^. Finally, for the comparison of SLE versus CRC patients, maximum sensitivity and specificity, 100% for both, were achieved at a cut-off of 2.45 ng mL^−1^ of BAFF.

In the case of APRIL, equivalent 100% sensitivity and specificity were achieved for the comparison of SLE and CRC patients against the healthy individuals for 4.3 ng mL^−1^ and 3.5 ng mL^−1^, respectively. However, no significant differences could be found between SLE and CRC patient APRIL levels.

## Conclusions

This work reports the first bioplatforms for the simultaneous determination of BAFF and APRIL, two relatively newly described cytokines of great relevance in autoimmune and cancer diseases. The immunoplatform, implemented in an integrated format, involved the use of *p*-aminobenzoic acid-grafted, screen-printed carbon electrodes to allow the efficient immobilization of the capture antibodies, and the use of MWCNTs decorated with MoS_2_ nanoparticles as nanocarriers of the detector antibodies and HRP to carry out the electrochemical signal amplification. An amperometric transduction at − 0.20 V (vs. Ag pseudo-reference electrode) using SPdCE in the presence of the hydroquinone/H_2_O_2_ system allowed an excellent sensitivity (LOD values of 0.08 and 0.06 ng mL^−1^ for BAFF and APRIL, respectively) to be achieved as well as a great selectivity, and operational and storage stability (cAb_BAFF/APRIL_-Neu-Phe-SPdCE ≥ 30 days; MWCNTs/MoS_2_-(HRP)-dAb_BAFF/APRIL_ ≥ 100 days). The dual immunoplatform allowed the accurate determination of both target cytokines in cancer cell lysates (0.5 μg/determination) and serum samples (10 times diluted) of patients diagnosed with SLE and CRC without matrix effect. These pioneering results demonstrate the potential of these dual immune platforms as well as their competitiveness against commercially available ELISA methodologies for the single determination of the target biomarkers, to be easily transferable to the clinic for their simplicity, affordable cost, reduced assay time, and point-of-care and multiplexed operation. These results open a new avenue to assist in the management of these patients and to further investigate the role played by these cytokines in these or other prevalent diseases.

## Supplementary Information

Below is the link to the electronic supplementary material.

Supplementary file1 (DOCX 559 KB)
