# Supplementary material for: Binary MoS2 nanostructures as nanocarriers for amplification in multiplexed electrochemical immunosensing: simultaneous determination of B cell activation factor and proliferation-induced signal immunity-related cytokines
Source: Mikrochim Acta. 2022 Mar 14;189(4):143. doi: 10.1007/s00604-022-05250-4 (PMC8921152; doi:10.1007/s00604-022-05250-4)
Supplement: Supplementary file 1 — Supplementary file1 (DOCX 559 KB) [file 604_2022_5250_MOESM1_ESM.docx]

**SUPPLEMENTARY INFORMATION**

**Binary MoS_2_ nanostructures as nanocarriers for amplification in multiplexed electrochemical immunosensing. Simultaneous determination of B cell activation factor and a proliferation-induced signal immunity-related cytokines**

Beatriz Arévalo^1^, Marina Blázquez-García^1^, Alejandro Valverde^1^, Verónica Serafín^1^, Ana Montero-Calle^2^, Guillermo Solís-Fernández^2^, Rodrigo Barderas^2^, Susana Campuzano^1,*^, Paloma Yáñez-Sedeño^1,*^, José M. Pingarrón^1^

^1^Department of Analytical Chemistry, Faculty of Chemistry, Complutense University of Madrid, 28040-Madrid. Spain.

^2^Chronic Disease Programme, UFIEC, Institute of Health Carlos III, Majadahonda, 28220-Madrid. Spain.

* to whom correspondence should be addressed

E-mails: [susanacr@quim.ucm.es](mailto:susanacr@quim.ucm.es); [yseo@quim.ucm.es](mailto:yseo@quim.ucm.es)

| **CONTENTS** | **PAGE** |
| --- | --- |
| Optimization of the experimental variables involved in the preparation of the dual immunosensor | S3-S8 |
| Effect of loadings and composition of MoS_2_/MWCNTs(-HRP)-dAbs | S3-S5 |
| Fig. S1 | S4-S5 |
| Selection between Strep or Neu protein; loading and incubation time of Neu onto Phe-SPdCEs | S5-S6 |
| Fig. S2 | S5-S6 |
| Effect of the cAb loading and incubation time | S6 |
| Fig. S3 | S6 |
| Effect of the type and concentration of the blocking agent and the corresponding incubation time | S6-S7 |
| Fig. S4 | S7 |
| Effect of the target and the carrier tag incubation time | S7-S8 |
| Fig. S5 | S8 |
| EIS characterization of immunosensors´ stepwise fabrication | S8-S9 |
| Fig. S6 | S8-S9 |
| Storage stability | S9 |
| Fig. S7 | S9 |
| Table S1 | S9-S10 |
| Fig. S8 | S10 |
| Table S2 | S10-S11 |
| Fig. S9 | S12 |
| References | S13 |

**Optimization of the experimental variables involved in the preparation of the dual immunosensor**

The effect of the experimental variables involved in the preparation of MoS_2_/MWCNTs(-HRP)-dAb_BAFF_ (or -dAb_APRIL_) carrier tags and the cAb_BAFF_-Neu-Phe-SPdCE (or cAb_APRIL_-) bioelectrodes, on the amperometric responses provided by the resulting MoS_2_/MWCNTs(-HRP)-dAb_BAFF_-BAFF-cAb_BAFF_-Neu-Phe-SPdCE or MoS_2_/MWCNTs(-HRP)-dAb_APRIL_-APRIL-cAb_APRIL_-Neu-Phe-SPdCE, was evaluated. Larger ratio between the currents measured with the as prepared immunosensors in the presence (S) of 5 ng mL^-1^ or 4 ng mL^-1^ BAFF or APRIL standards, respectively, or in the absence (B) of the target compounds was taken as the selection criterion for each tested variable. The optimization studies implied the evaluation of: a) MoS_2_/MWCNTs(-HRP)-dAb_BAFF_ (or -dAb_APRIL_) loadings, and those of dAb_BAFF_ (or dAb_APRIL_) and HRP onto the MoS_2_/MWCNTs nanocomposite; b) selection between Strep or Neu binding protein immobilized onto Phe-SPdCEs; c) loading and incubation time of Neu onto Phe-SPdCEs; d) concentration and incubation time of biotinylated capture antibodies (cAbs) onto Neu-Phe-SPdCEs; e) type, concentration and incubation time of the blocking agent; f) incubation time of BAFF or APRIL cytokines onto cAb-Neu-Phe-SPdCEs; g) incubation time of the corresponding nanocarrier tag onto BAFF-cAb_BAFF_-Neu-Phe-SPdCE or APRIL-cAb_APRIL_-Neu-Phe-SPdCE. The results of these studies are shown in the Supplementary Material (Figs. S1-S5).

**Effect of loadings and composition of MoS_2_/MWCNTs(-HRP)-dAbs**

Figure S1 shows the results obtained in the optimization studies of the loading and composition of the carrier tags. An increase in the amount of MoS_2_/MWCNTs(-HRP)-dAb_BAFF_ (Fig. S1a) gave rise to a slight increase in the blank signal, reaching a larger S/B ratio for 0.1 mg mL^-1^. In the case of APRIL (Fig. S1d), a continuous increase both in the response of the immunosensor in the presence of the antigen and in the blank signal was observed, reaching a larger S/B ratio for 0.2 mg mL^-1^.

Regarding the effect of the dAbs loading onto the carrier tag on the response of the respective immunosensor (Figs. S1b and e), in both cases, as expected, an increase in the responses in the presence of the target cytokine was observed as the amount of the respective dAb increased until reaching the saturation concentration. Furthermore, blanks remained practically constant over the tested range. Accordingly, 6 μg mL^-1^ and 5 μg mL^-1^ were selected for the preparation of BAFF and APRIL immunosensors, respectively.

The effect of the HRP loading immobilized onto the carrier tag on the immunosensors response is shown in Figures S1c and f. In the case of BAFF, as expected, the specific current increased with the HRP concentration and, more slightly, also the blank signal, reaching a larger S/B ratio for 1 mg mL^-1^. However, the APRIL immunosensor exhibited an abnormal behaviour with very large specific and blank currents for concentrations above 1 mg mL^-1^. Therefore, this concentration was chosen to prepare both immunosensors.

**Fig. S1** Effect of: (a d) MoS_2_/MWCNTs(-HRP)-dAb nanocomposites loadings, (b, e) dAb and (c, f) HRP loadings onto MoS_2_/MWCNTs(-HRP)-dAb on the amperometric responses of: MoS_2_/MWCNTs(-HRP)-dAb_BAFF_-BAFF-cAb_BAFF_-Phe-SPCE or MoS_2_/MWCNTs(-HRP)-dAb_APRIL_-APRIL-cAb_APRIL_-Phe-SPCE immunosensors for 0 (white bars, B) and 5 ng mL^-1^ BAFF or 4 ng mL^-1^ APRIL (grey bars, S) standards and the corresponding S/B ratio values (in red).

Electrochemical platforms: 10 μL Strep 600 μg mL^-1^, 45 min; 5 μL cAb_BAFF_ 100 ng mL^-1^ or cAb_APRIL_  5 μg mL^-1^, 60 min; 10 μL 2 % casein + 3 mg mL^-1^ biotin (BAFF) or 2 mg mL^-1^ biotin (APRIL), 60 min.

Carrier tags: 5 μL MoS_2_/MWCNTs(-HRP)-dAb_BAFF_ , 60 min; 6 μg mL^-1^ dAb_BAFF_ ;1 mg mL^-1^ HRP (a); 5 μL MoS_2_/MWCNTs(-HRP)-dAb_BAFF_ 0.1 mg mL^-1^, 60 min ; 1 mg mL^-1^ HRP (b); 5 μL MoS_2_/MWCNTs(-HRP)-dAb_BAFF_ 0.1 mg mL^-1^ , 60 min; 6 μg mL^-1^ dAb_BAFF_ (c); 5 μL MoS_2_/MWCNTs(-HRP)-dAb_APRIL_ , 30 min; 10 μg mL^-1^ dAb_APRIL_; 1 mg mL^-1^ HRP (d); 5 μL MoS_2_/MWCNTs(-HRP)-dAb_APRIL_ 0.2 mg mL^-1^, 30 min; 1 mg mL^-1^ HRP (e); 5 μL MoS_2_/MWCNTs(-HRP)-dAb_APRIL_ 0.2 mg mL^-1^, 60 min; 5 μg mL^-1^ dAb_APRIL_ (f); 1 mM HQ; 50 mM H_2_O_2_ ; E_app_ = -0.20 V (vs. Ag pseudo-reference electrode). See text for more details. Error bars estimated as triple of the standard deviation (n = 3)

**Selection between Strep or Neu protein; loading and incubation time of Neu onto Phe-SPdCEs**

The immunosensors responses obtained using Strep or Neu as binding proteins of biotinylated capture antibodies are compared in Fig. S2a and d, for BAFF and APRIL, respectively. As it can be observed, both immunosensors exhibited a similar behaviour with Neu providing larger S/B ratios. This behaviour agrees with that reported previously [1] and can be attributed to the nearly neutral charge of Neu that minimizes nonspecific protein-protein interactions [2]. The Neu loading and incubation time effect on the amperometric responses is displayed in Figs. S2b-d. The BAFF immunosensor (Fig. S2 b) provided currents that rapidly increased with Neu concentration up to 600 μg mL^-1^, then decreasing probably as a consequence of a blocking effect on the electrode surface. A similar trend was found in the case of APRIL immunosensor, although the blank current gradually increased with Neu concentration and the blocking effect was apparent at smaller concentrations. According to these results into account, a Neu concentration of 600 μg mL^-1^ was chosen to prepare the BAFF immunosensor, and of 400 μg mL^-1^ for the APRIL immunosensor. Furthermore, Neu incubation times on the Phe-SPdCEs electrodes of 15 and 30 min (Figs, S2 c and f) were selected.

**Fig. S2** Effects on the responses of BAFF (a,b,c) and APRIL (d,e,f) immunosensors of: the use of Strep or Neu as binding proteins for the biotinylated cAbs (a,d); loading (b,e) and immobilization time (c,f) of Neu onto Phe-SPdCEs. See the text and Fig S1 for more information

**Effect of the cAb loading and incubation time**

These studies provided similar trends for both biomarkers (Fig. S3). Regarding the influence of the cAb concentration immobilized onto Neu-Phe-SPdCEs (Figs. S3a and c), nearly constant currents were obtained from 50 ng mL^-1^ BAFF or 2.5 ng mL^-1^ APRIL. Larger S/B ratios were found for 100 and 5 ng mL^-1^ respectively, these concentrations being selected for further work. Furthermore, incubation times of 30 (BAFF) and 15 (APRIL) min were selected (Figs. S3b and d).

**Fig. S3** Effect of the cAb loading (a,c) and incubation time (b,d) on the immunosensors response. See the text and Figure S1 for more information

**Effect of the type and concentration of the blocking agent and the corresponding incubation time**

The effect of the use of biotin as blocking agent and mixture solutions with other blocking agents (casein and BSA) on the immunosensors responses was checked (Figs. S4a and d). Larger S/B ratios were obtained in both cases using only biotin as the blocking agent. In addition, a better blocking behaviour was observed using 2 mg mL^-1^ biotin (Figs. S4b and e). Larger concentrations provoked a current decrease probably due to the lower conductivity of the electrode. Moreover, Figs. S4c and f show as an incubation time of 15 min was enough to obtain a good blocking of the electrode surfaces with longer times causing a decrease in the S/B ratios.

**Fig. S4** Effect of the type (a,c), concentration (b,d) and incubation time (c,f) of the used blocking agent on the immunosensors responses. A) 2 mg mL^-1^ biotin; B) 2% casein + 2 mg mL^-1^ biotin; C) 2% BSA; D) 2% casein + 1% BSA + 3 mg mL^-1^ biotin. See the text and Figure S1 for more information

**Effect of the target and the carrier tag incubation time**

The effect of the incubation time of each target biomarker on the cAb-Neu-Phe-SPdCE conjugate was evaluated. Results are shown in Figure S5 (a and c). The S/B ratio varied in a similar manner for both immunosensors, with 30 min providing larger ratios. The responses of the immunosensors showed a similar behaviour with the incubation time of the carrier tag (Figs. S5 b and d). 30 (BAFF) and 15 (APRIL) min were chosen for further work.

**Fig. S5** Effect of the incubation times of BAFF (a) and APRIL (c), and the respective carrier tag (b, BAFF and d, APRIL) on the immunosensors responses. See the text and Figure S1 for more information

**EIS characterization of immunosensors´ stepwise fabrication**

**Fig. S6** Nyquist curves and equivalent circuits recorded at the different steps involved in the BAFF (a, b) and APRIL (c, d) immunosensors preparation. 5 mM [Fe(CN)_6_]^3-/4-^ in 0.1 M KCl solution as redox probe, 0.04−100,000 Hz frequency range with a 10 mV r.m.s. signal.

**Storage stability**

**
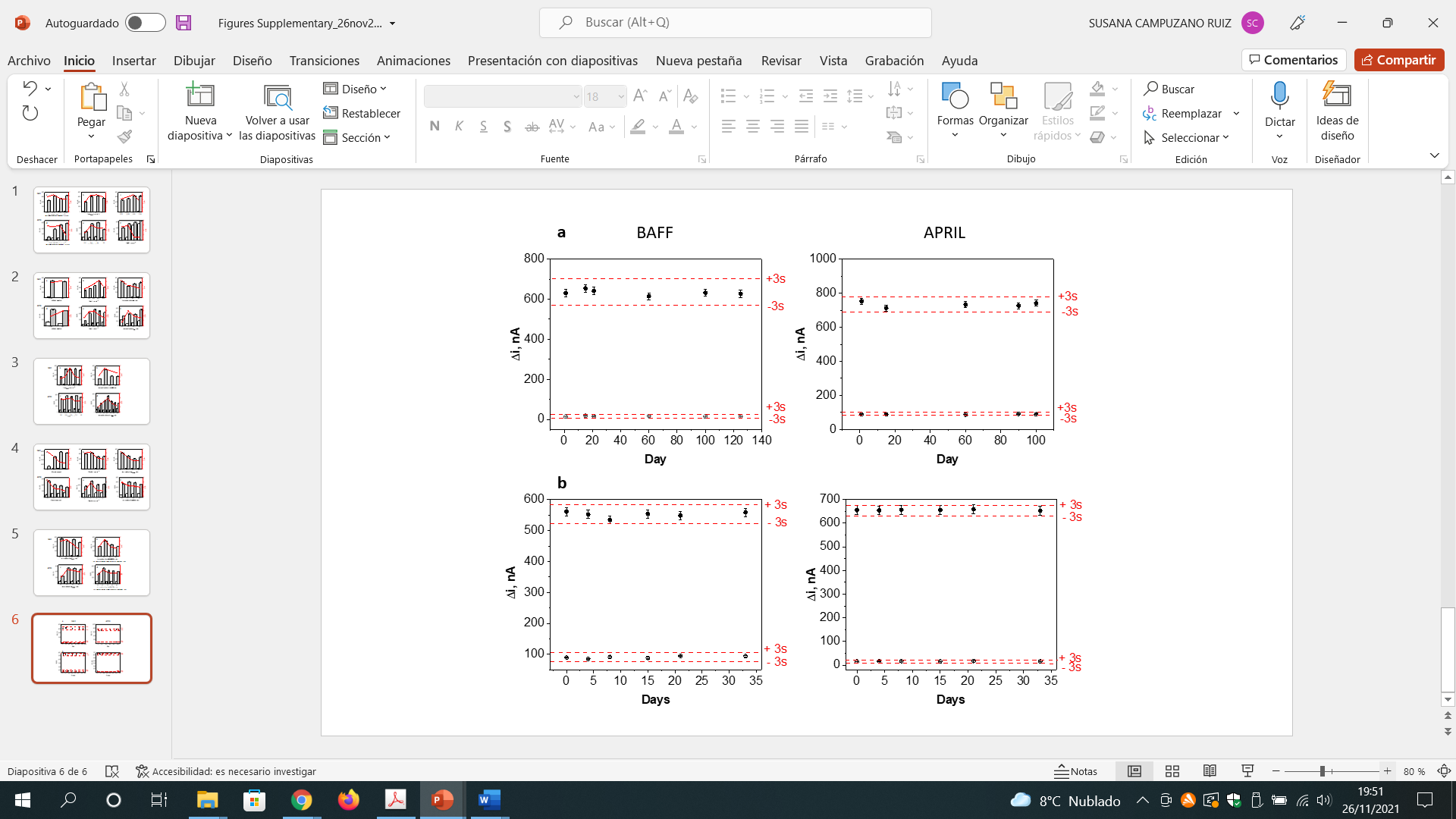
**

**Fig. S7** Storage stability of the MWCNTs/MoS_2_-(HRP)-dAb_BAFF/APRIL_ bionanoconjugates (a) and cAb_BAFF/APRIL_-Neu-Phe-SPdCE immunoplatforms (b)

**Table S1.** Δi values (in nA) corresponding to bars displayed in Figures 9a) and b).

|  | BAFF | | APRIL | |
| --- | --- | --- | --- | --- |
|  | Blank | Signal | Blank | Signal |
| No Interferent | 14.9 | 734.7 | 422.9 | 1646 |
| IgG | 14.7 | 768.2 | 445.6 | 1659 |
| HSA | 15.0 | 761.9 | 455.5 | 1684 |
| BSA | 14.8 | 751.5 | 461.8 | 1722 |
| HB | 15.1 | 708.8 | 527.4 | 1881 |
| NfL | 14.8 | 751.5 | 435.3 | 1686 |
| Tau | 15.0 | 724.9 | 512.1 | 1929 |
| TDP-43 | 14.7 | 742.4 | 542.4 | 2013 |
| CDH-17 | 14.9 | 754.9 | 545.4 | 1919 |
| IL13Rα2 | 15.1 | 758.4 | 522.7 | 1982 |
| TNFα | 15.3 | 736.8 | 578.4 | 2020 |
| APRIL/ BAFF | 14.9 | 773.1 | 558.8 | 1965 |

| Mixture | 0/0 | 25/0 | 0/100 | 25/100 |
| --- | --- | --- | --- | --- |
| APRIL | 91.7 | 758.4 | 95.5 | 745.4 |
| BAFF | 12.1 | 14.6 | 959.7 | 969.7 |

**Fig. S8** Simultaneous amperometric responses (and corresponding Δi values, in nA, in the Table) measured with the dual immunosensors at the WE1 (BAFF, grey bars) and WE2 (APRIL, white bars) and in standard mixtures containing the indicated concentration of both target cytokines.

**Table S2** Comparison between the slope values (in nA per decade of BAFF/APRIL concentration) of the calibration plots constructed with the dual immunosensor for standards prepared in buffer and in representative 10-fold diluted serum and 0.5 µg of cells extract samples

| Matrix | BAFF | t_exp_^*^ | APRIL | t_exp_^*^ |
| --- | --- | --- | --- | --- |
| Buffered solutions | 244 ± 3 | -- | 258 ± 5 | -- |
| 10-times diluted serum | 230 ±8 | 1.638 | 248 ± 3 | 1.715 |
| 0.5 μg cells extract | 233 ± 6 | 1.639 | 261 ± 17 | 0.169 |

*^*^t_exp_ estimated by comparing the slope obtained for standards prepared in the corresponding matrix and in buffered solutions, t_tab_ = 2.776, n = 4, α= 0.05.*

**Fig. S9** ROC curves of the diagnostic value for the discrimination of SLE and CRC patients from controls​. The determination of BAFF (a) and APRIL (b) levels in serum samples showed a high diagnostic ability of SLE and CRC patients in comparison with healthy individuals, with an AUC, sensitivity and specificity of 100%. (c) BAFF levels in serum samples possesed a high capacity for the discrimination of SLE and CRC patients, whereas the determination of APRIL levels in serum samples reduced the discriminatory capacity of SLE and CRC patients to 75%, with a sensitivity and specificity of 75% and 100%, respectively.

**References**

1. Arévalo B, Serafín V, Sánchez-Paniagua M, Montero A, Barderas R, López-Ruíz B, Campuzano S, Yáñez-Sedeño P, Pingarrón JM (2020) Fast and sensitive biosensor for diagnosis of autoimmune disorders through amperometric determination of serum anti-dsDNA autoantibodies. Biosens Bioelectron 160:112233

2. Jain A, Barve A, Zhao Z, Jin W, Cheng K (2017) Comparison of avidin, neutravidin, and streptavidin as nanocarriers for efficient siRNA Delivery. Mol Pharm 14:1517−1527
